# Targeting Protein *O*-GlcNAcylation, a Link between Type 2 Diabetes Mellitus and Inflammatory Disease

**DOI:** 10.3390/cells11040705

**Published:** 2022-02-17

**Authors:** Israel Olapeju Bolanle, Timothy M. Palmer

**Affiliations:** Centre for Atherothrombosis and Metabolic Disease, Hull York Medical School, University of Hull, Hull HU6 7RX, UK; hyob4@hyms.ac.uk

**Keywords:** *O*-GlcNAcylation, metabolism, diabetes, inflammation

## Abstract

Unresolved hyperglycaemia, a hallmark of type 2 diabetes mellitus (T2DM), is a well characterised manifestation of altered fuel homeostasis and our understanding of its role in the pathologic activation of the inflammatory system continues to grow. Metabolic disorders like T2DM trigger changes in the regulation of key cellular processes such as cell trafficking and proliferation, and manifest as chronic inflammatory disorders with severe long-term consequences. Activation of inflammatory pathways has recently emerged as a critical link between T2DM and inflammation. A substantial body of evidence has suggested that this is due in part to increased flux through the hexosamine biosynthetic pathway (HBP). The HBP, a unique nutrient-sensing metabolic pathway, produces the activated amino sugar UDP-GlcNAc which is a critical substrate for protein *O*-GlcNAcylation, a dynamic, reversible post-translational glycosylation of serine and threonine residues in target proteins. Protein *O*-GlcNAcylation impacts a range of cellular processes, including inflammation, metabolism, trafficking, and cytoskeletal organisation. As increased HBP flux culminates in increased protein *O*-GlcNAcylation, we propose that targeting *O*-GlcNAcylation may be a viable therapeutic strategy for the prevention and management of glucose-dependent pathologies with inflammatory components.

## 1. Introduction

Sustained hyperglycaemia, which is the hallmark of type 2 diabetes mellitus (T2DM), comes with far reaching consequences such as increased risk of cardiovascular diseases (CVD) and kidney failure [1]. While some of the complications associated with hyperglycaemia are the result of acute metabolic derangements e.g., ketoacidosis, a majority of complications are due in part to chronically elevated blood glucose levels e.g., stroke, neuropathy, retinopathy and nephropathy [1]. Metabolic alterations associated with hyperglycaemia have been well characterised [2]. Specifically, the pathogenesis and progression of T2DM have been ascribed to four key mechanisms; increased polyol pathway flux, increased advanced glycation end product (AGE) formation, activation of protein kinase C (PKC) isoforms, and increased hexosamine pathway flux [2,3]. However, until recently, the contribution of increased hexosamine pathway flux to the development of metabolic disorders was unclear [2,4,5,6]. The final product of the hexosamine biosynthetic pathway (HBP) is the activated amino sugar UDP-GlcNAc, a critical substrate for protein glycosylation [6,7,8]. UDP-GlcNAc serves as the sugar donor for *O*-GlcNAcylation events which is a dynamic, reversible post-translational modification of serine and threonine residues in target proteins within cells [8,9]. This process is controlled by two enzymes: *O*-linked β-*N*-acetylglucosamine transferase (OGT) and *O*-linked β-*N*-acetylglucosaminidase (OGA) [8,9]. OGT is responsible for catalysing the addition of a *O*-GlcNAc moiety to either serine or threonine residues in target proteins [10,11]. Conversely, the enzyme OGA reverses this modification by catalysing the hydrolysis of *O*-GlcNAc from protein targets [10,11]. A large body of evidence has shown that increased hexosamine pathway flux results in increased OGT-mediated *O*-GlcNAcylation events [6,12,13,14,15]. Importantly, *O*-GlcNAcylation of inflammatory pathways, and trafficking and proliferation of cells have emerged as a critical link between T2DM and the chronic inflammation observed in pathologies that are a consequence of poor blood glucose control [2,4,5,16]. In recent years, a growing number of findings elucidating the role of *O*-GlcNAcylation as vital molecular mechanism in the pathogenesis of T2DM and its complications have emerged [2,4,5,12,13,14,15,16]. In this review, we will, therefore, evaluate the involvement of *O*-GlcNAcylation in the inflammatory events induced by T2DM and the potential therapeutic viability of targeting *O*-GlcNAcylation in preventing and managing disease progression.

## 2. Hexosamine Biosynthetic Pathway (HBP) and *O*-GlcNAcylation

### 2.1. Hexosamine Biosynthetic Pathway (HBP)

The HBP is a distinct nutrient-sensing metabolic pathway that produces the activated amino sugar UDP-*N*-acetyl-glucosamine (UDP-GlcNAc), a key substrate for protein glycosylation. This type of glycosylation is particularly sensitive to changes in UDP-GlcNAc concentration. However, HBP and *O*-GlcNAcylation are not only sensitive to changes in glucose concentrations but also changes in protein and lipid metabolism [17]. Over the years, there have been significant advances in our understanding of this dynamic pathway. An important breakthrough came when Marshall et al. [18] treated isolated rat adipocytes with glucosamine (GlcN) to demonstrate the contribution of glucose flux through the HBP to the development of insulin resistance. Given the likelihood that glucose and GlcN mediate desensitisation through the same mechanism and, since GlcN appears to be 40 times more potent than glucose, it was suggested that an estimated 2–3% of the incoming glucose that is converted to fructose-6-phosphate (Fru-6-P) enters the HBP [18]. However, recent findings by Olsen et al. [19] using a more reliable quantitative assay in an ex vivo mouse heart model showed that glucose metabolism through the HBP, as determined by the rates of glycolysis and UDP-GlcNAc synthesis, comprises only ~0.006% of the glycolytic efflux, which is much lower than that originally proposed [18]. The 2–3% estimate from Marshall et al. [18] was from a single study in rat adipocyte cultures and expressed as a representation of the percentage of total glucose uptake. However, it is unclear whether this value would be consistent between quiescent cells in culture versus metabolically active organs with high energy demand such as the heart. In view of these shortcomings, the estimate by Olsen et al. [19] may be more quantitatively reliable.

**Figure 1:**
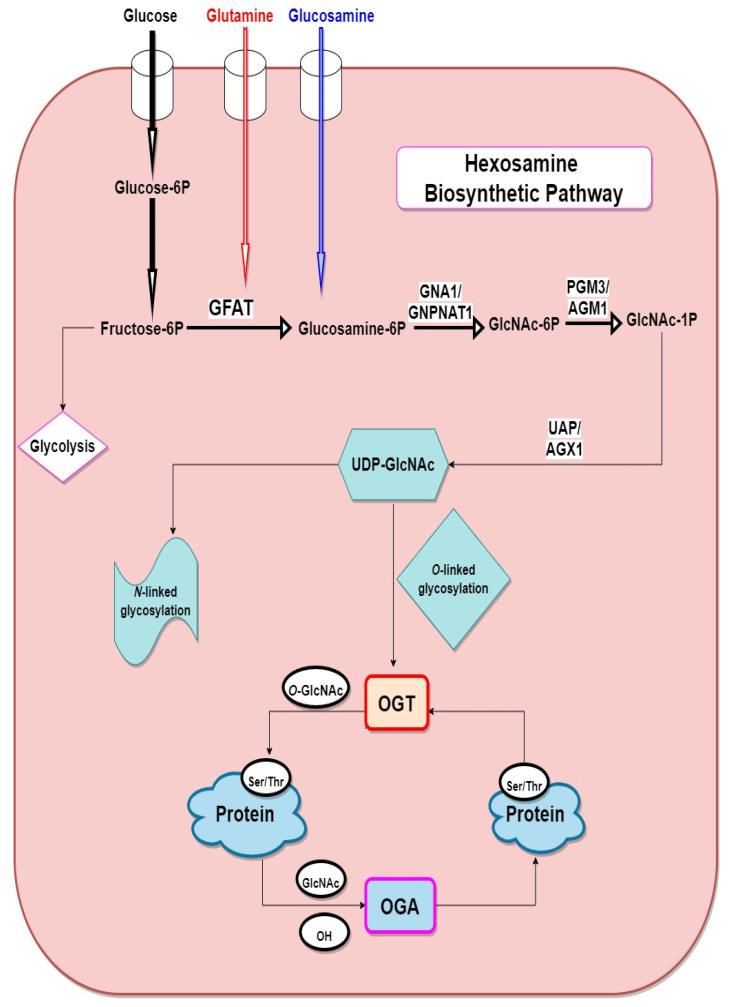
Schematic of the hexosamine biosynthetic pathway. fructose-6P (fructose-6-phosphate), glucosamine-6P (glucosamine-6-phosphate), GFAT (glutamine:fructose-6-phosphate amidotransferase), OGT (*O*-GlcNAc transferase), OGA (*O*-GlcNAcase) GNA1/GNPNAT1 (glucosamine-6-phosphate *N*-acetyltransferase), GlcNAc-6P (*N*-acetylglucosamine-6-Phosphate), GlcNAc-1P (*N*-acetylglucosamine-1-phosphate), PGM3/AGM1 (phosphoglucomutase), UDP-GlcNAc (uridine diphosphate-*N*-acetylglucosamine), UAP/AGX1 (UDP-*N*-acetylhexosamine pyrophosphorylase).

As shown in Figure 1, when glucose enters the cell, it is converted to fructose-6P (fructose-6-phosphate), following this, glutamine-fructose-6P amidotransferase 1 (GFAT), the rate-limiting enzyme in the HBP transfers an amino group to fructose-6-phosphate from glutamine to form glucosamine-6-phosphate (GlcN-6-P). GlcN-6P is then rapidly acetylated by glucosamine-phosphate *N*-acetyltransferase (GNPNAT, EMeg32) in the presence of Acetyl-CoA, to produce *N*-acetylglucosamine-6-phosphate (GlcNAc-6P) [20], which further undergoes isomerisation by GlcNAc phosphomutase (PGM3/AGM1) to produce *N*-acetylglucosamine-1-phosphate (GlcNAc-1P) [21]. Then the nucleoside is added to the sugar by UDP-*N*-acetylhexosamine pyrophosphorylase 1 (UAP/AGX1) to yield UDP-GlcNAc which is the amino sugar substrate. UDP-GlcNAc is then used as a substrate for *N*- and *O*-linked glycosylation reactions in the ER and Golgi and for *O*-GlcNAc modification of nuclear and cytoplasmic proteins by OGT (*O*-GlcNAc transferase). OGA (*O*-GlcNAcase) catalyses the removal of *O*-GlcNAc and adds back GlcNAc to the HBP pool for re-cycling through the salvage pathway. Studies have shown that unresolved and sustained hyperglycaemia increases glycolytic efflux and subsequent activation of HBP which then increases production of the amino sugar substrate UDP-GlcNAc for protein glycosylation [6,12,13,14,15].

### 2.2. Protein O-GlcNAcylation

**Figure 2:**
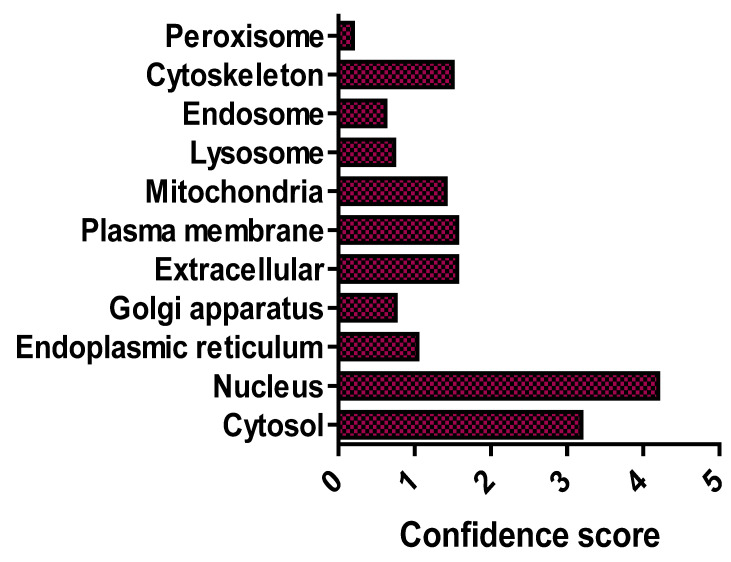
Subcellular distribution of the *O*-GlcNAcome. Median confidence scores of human *O*-GlcNAcylated proteins (*n* = 4969) are from [22].

*O*-GlcNAcylation is a dynamic post-translational glycosylation that links single GlcNAc molecules to target serine and threonine residueson target proteins by an *O*-linked β-glycosidic bond [21]. The *O*-GlcNAcome recently reached a milestone of 5000 human proteins identified [22]. Distinct from other protein glycosylation events, which are chiefly produced by the secretory pathways, *O*-GlcNAcylated proteins are predominantly localised in the nucleus and cytoplasm, with only approximately 7% of *O*-GlcNAc moieties detectable at the cell surface [7,21]. Furthermore, cellular distribution analysis has confirmed that *O*-GlcNAcylated proteins are chiefly concentrated in nuclear and cytoplasmic compartments (Figure 2) [22]. *O*-GlcNAcylation has been shown to be highly enriched on proteins in nuclear pore complexes and the nuclear envelope [21,23], as well as proteins that can interact with chromatin [24]. Cytoskeletal [25], and intrinsic membrane proteins in the Golgi apparatus and endoplasmic reticulum (ER) have also been shown to be *O*-GlcNAcylated [26]. Unlike phosphorylation and other post-translational modifications (PTMs), there is currently no consensus sequence that can accurately predict target protein modification by *O*-GlcNAcylation.

As stated earlier, a single pair of enzymes—OGT and OGA—controls this dynamic cycling [15,16]. OGT catalyses the attachment of a monosaccharide (GlcNAc) moiety to the free hydroxyl group of either serine or threonine residues in target proteins through an *O*-glycosidic linkage [10,11,27]. The human *OGT* gene produces three known isoforms of OGT namely the ncOGT, mOGT, and sOGT. The ncOGT which is the longest of the isoforms is localized to both the nucleus and cytoplasm. It contains a unique *N*-terminal sequence followed by 12 tetratricopeptide repeats (TPR) motifs, a linker region, and the catalytic domain. Furthermore, mOGT, the second isoform has a distinct *N*-terminal sequence which includes a mitochondrial targeting motif. The *N*-terminal sequence is then followed by 9 TPR motifs, a linker region, and the catalytic domain. sOGT, the shortest of the isoforms is ubiquitously expressed. Characteristically, it has only 2 TPR motifs, a linker region, and the catalytic domain. Importantly, all three isoforms have identical catalytic region which contained two sub-domains termed CD I and CD II [11,27].

Conversely, OGA, which was first purified from rat spleen cytosol, reverses *O*-GlcNAcylation by catalysing the hydrolysis of *O*-GlcNAc from protein targets [11]. OGA is largely enriched in the cytosol, unlike OGT, which accumulates in the cytoplasm and nucleus [28]. The OGA transcript encodes a protein with three distinct regions: a *N*-terminal catalytic domain, a stalk domain and a C-terminal pseudo-histone acetyltransferase domain [29,30]. The human OGA can exist as a homodimer with a unique interaction with other OGAs. During this interaction, it uses the substrate-recognition mode in which its stalk domain combines with the catalytic domain from other OGA monomer, and this interaction forms a cleft for substrate binding [30,31,32,33]. Also, hydrophobic residues are chiefly dominant in the inner surface of the substrate binding cleft, and are conserved in most eukaryotes. These hydrophobic interactions are important for protein binding and spatial constraints [30].

## 3. Involvement of *O*-GlcNAcylation in Inflammation

### 3.1. Impact of O-GlcNAcylation on Inflammatory Signalling Pathways

Activation of inflammatory signalling pathways by glucose-dependent metabolic stress is a major phenomenon in the pathogenesis and progression of T2DM [2,4] as well as other known pathologies such as cancers, autoimmune disorders and neurological disease [34,35,36,37,38]. Despite our incomplete understanding of the mechanisms responsible for nutrient regulation of inflammation, several studies suggest that *O*-GlcNAcylation is critical to the pathophysiology of inflammatory derangements [37,39,40,41,42,43]. Importantly, while some studies have indicated that *O*-GlcNAcylation of target proteins is a pro-inflammatory event in diabetes and is involved in the development of insulin resistance [44,45]. Conversely, others have shown that this dynamic post-translational modification can also confer protection against acute inflammatory stimuli [46,47,48]. These findings [44,45,46,47,48] strongly suggest that diverse forms of insults could result in dynamic changes to *O*-GlcNAcylation patterns to either promote or inhibit inflammation in response to fluctuations in cellular metabolism. This section of the review highlights core involvement of *O*-GlcNAc modification of target proteins in inflammatory pathways.

#### 3.1.1. Pro-Inflammatory Role of *O*-GlcNAcylation

**Figure 3:**
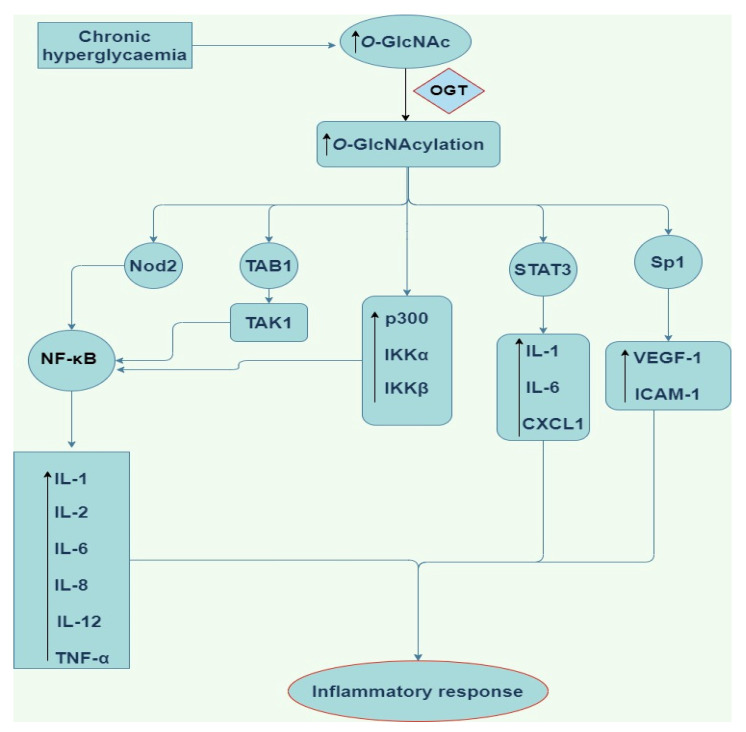
Schematic of the pro-inflammatory downstream signalling modulation by *O*-GlcNAcylation. *O*-GlcNAc (*O*-linked β-*N*-acetylglucosamine), OGT (*O*-linked β-*N*-acetylglucosamine transferase), NF-κB (nuclear factor kappa light chain enhancer of activated B cells), STAT3 (signal transducer and activator of transcription3), TAK 1 (Transforming growth factor-β activated kinase 1), TAB1 (TAK1-binding protein 1), Nod2 (nucleotide-binding oligomerization domain-containing protein 2), IL-1,6,8, and 12 (Interleukin-1,6,8, and 12), Sp1 (substrate of Keap1), CXCL1 (chemokine ligand 1), VEGF-A (vascular endothelial growth factor), ICAM-1 (intercellular Adhesion Molecule 1), TNF-α (tumor necrosis factor alpha).

Emerging findings have continued to improve our understanding of the pro-inflammatory role of *O*-GlcNAcylation in the NF-κB signaling pathway. NF-κB is a chief regulator of innate and adaptive immune responses and plays a central role in chronic inflammation responsible for tumour development, rheumatoid arthritis, atherosclerosis, chronic obstructive pulmonary disease, asthma, multiple sclerosis, inflammatory bowel disease, and ulcerative colitis [49,50,51,52,53]. Yang et al. [54] have shown that the p65/RelA NF-κB subunit is *O*-GlcNAcylated in rat vascular smooth muscle cells. *O*-GlcNAc modification of NF-κB p65 on Thr352 inhibits the interaction between NF-κB p65 and IκB, thereby promoting translocation of NF-κB heterodimers to the nucleus and increasing transcription of target genes such as the gene for adhesion molecule VCAM-1 under hyperglycaemic conditions [50]. These findings strongly suggest that specific *O*-GlcNAcylation of p65/RelA on Thr352 may facilitate sustained activation of NF-κB-mediated chronic inflamamtion in response to prolonged hyperglycaemia and development of diabetes [50]. Although the mechanism of how *O*-GlcNAcylation activates NF-κB signaling is not fully understood, however, *O*-GlcNAcylation has been shown to increase the phosphorylation and acetylation of NF-κB subunit p65/RelA [51]. *O*-GlcNAc modification of p65 at Thr305 and Ser319 increased CREB-binding protein (CBP)/p300-dependent activating acetylation of p65 at Lys310, and this contributes to NF-κB transcriptional activation [51]. Also, elevation of *O*-GlcNAcylation by upregulating the expression of OGT increased the expression of p300, IKKα, and IKKβ and promoted IKK-mediated activating phosphorylation of p65 at Ser536, contributing to NF-κB activation (Figure 3) [51]. Similarly, it has been proposed that *O*-GlcNAcylation of IKKβ at Ser733 enhances NF-κB activity by increasing IKK activity, IκB phosphorylation and subsequent IκB degradation in both mouse and human fibroblasts [52]. These findings [49,50,52] suggest mechanisms through which *O*-GlcNAcylation regulates NF-κB signaling through interplay with phosphorylation and acetylation with consequent inflammatory outputs.

Furthermore, Sp1, a zinc finger transcription factor is also *O*-GlcNAcylated in response to high glucose concentrations and elevated Sp1 activity upon *O*-GlcNAcylation could play a role in hyperglycaemia-induced pro-fibrotic and pro-inflammatory factors involved in diabetic retinopathy [55,56]. Donovan et al. [55] showed that hyperglycaemia significantly increased Sp1 binding to the gene promoter of the pro-angiogenic mediator vascular endothelial growth factor (VEGF)-A, while shRNA-mediated knockdown of either OGT or Sp1 significantly inhibited glucose-induced increases in VEGF-A levels in ARPE-19 human retinal pigment epithelial cells and TR-iBRB rat retinal microvascular endothelial cells. These observations suggest that hyperglycaemia-induced *O*-GlcNAcylation of Sp1 drives VEGF-A production in both systems. Zhang et al. [56] have also proposed that hyperglycaemia also stimulates expression of intercellular adhesion molecule (ICAM)-1 by *O*-GlcNAcylation of Sp1 in both human umbilical vein endothelial cells and rat retinal capillary endothelial cells.

Alternatively, the transcriptional activity of Sp1 can be reduced when *O*-GlcNAc modified, and this may be due to the resultant distortion in its interaction with its cooperative factors such as Elf-1 [57], NF-Y [58], Ying-Yang 1 [59], and sterol regulatory element binding protein 2 [60]. Thus, *O*-GlcNAc modification of Sp1 could contribute to negative regulation of placental and embryonic expression of oncofetal protein gene (Pem), a gene which encodes a homeobox protein expressed in reproductive tissues and a wide range of transformed cells [57]. Furthermore, *O*-GlcNAcylation of Sp1 inhibits hyaluronan synthesis (an important component of the extracellular matrix that plays a crucial role for cumulus cell expansion which occurs during maturation of oocytes [59,60], and lipid synthesis [61]. Intriguingly, Suh et al. [62] demonstrated that *O*-GlcNAc modification of Sp1 protects primary cultured renal proximal tubule cells against hypoxia-induced dysfunction of Na/glucose cotransporter (SGLT). Similarly, Lee et al. [63] showed that *O*-GlcNAcylation of Sp1 protects mouse embryonic stem cells against hypoxia-induced apoptosis. A weakened association between Sp1 and its co-operative factors caused by *O*-GlcNAcylation is one potential mechanism which could explain these phenomena.

As a critical effector of cytokines which activate the Janus kinase (JAK)-STAT pathway, STAT3 function is tightly controlled [64]. However, our understanding of how metabolic changes regulate STAT3 function via specific PTMs that regulate STAT3 function, and their pathophysiological significance remain incomplete. Li et al. [46] demonstrated that there was an increased disease severity in azoxymethane-induced colitis and a colitis-associated cancer model in the bone marrow-derived macrophages isolated from CUL3-deficient mice. Li et al. [46] proposed that this was due to increased *O*-GlcNAcylation of STAT3. In addition, the expression levels of pro-inflammatory cytokines IL-1 and IL-6, and chemokines CXCL1 and CXCL2 were upregulated by *O*-GlcNAcylated STAT3 in BMMs from CUL3-deficient mice, thus contributing to azoxymethane-induced colitis and colitis-associated cancer.

Apart from the transcription factors highlighted above, other proteins which control inflammation and immunity e.g., Transforming growth factor-β activated kinase (TAK) 1 are also *O*-GlcNAcylated. TAK1 is an important serine/threonine protein kinase that mediates signals transduced by multifunctional cytokines such as TGF-β, TNF-α and IL-1 [65]. Emerging data have suggested that *O*-GlcNAcylation of TAK1-binding protein 1 (TAB1) at Ser395 is needed for TAK1 activation [65]. More so, activated TAK1 is intricate to downstream activation of NF-κB and production of IL-6 and TNF-α in human embryonic kidney (HEK) 293 cells stably expressing the IL-1 receptor and reconstituted *Tab1*-deficient MEFs upon exposure to IL-1 or hyperosmotic stress [65]. Upon stimulation, *O*-GlcNAc modification of TAK1 at Ser427 is needed for the phosphorylation of Thr187/Ser192 and full activation of TAK1 in RAW264.7 cells [66]. Thus, *O*-GlcNAc modification of TAK1 has been shown to trigger downstream activation of JNK and NF-κB signaling pathways, facilitate M1 polarisation of macrophages, and increase production of pro-inflammatory cytokine, resulting in the development of acute inflammatory responses [67]. Also, a cytoplasmic human nucleotide-binding oligomerization domain-containing protein 2 (Nod2)-like receptor that recognises bacterial components, is *O*-GlcNAcylated and this modification causes it to produce various pro-inflammatory cytokines and chemokines via activation of NF-κB in HEK293T cells (Figure 3) [42].

#### 3.1.2. Anti-Inflammatory Role of *O*-GlcNAcylation

**Figure 4:**
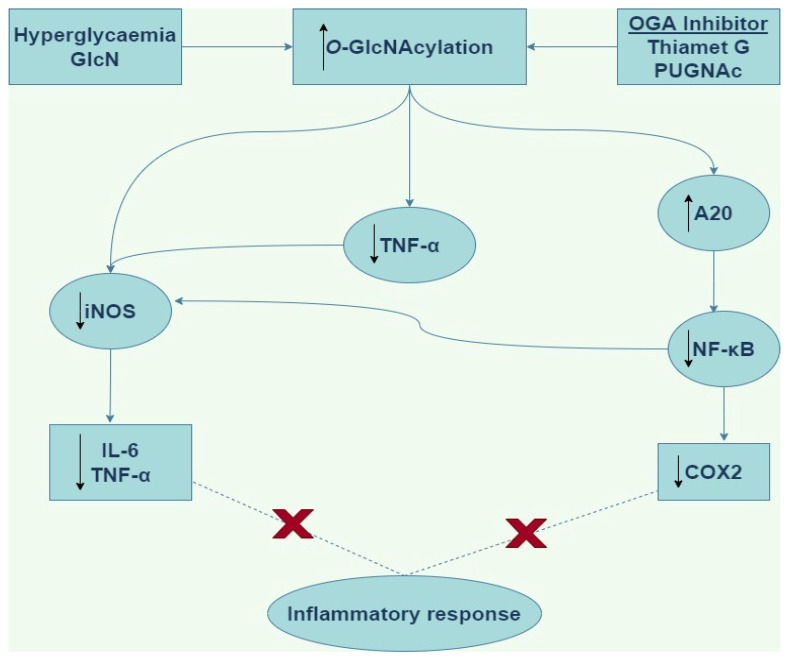
Schematic of the anti-inflammatory downstream signaling by *O*-GlcNAcylation. GlcN (Glucosamine), OGA (*O*-linked β-*N*-acetylglucosaminidase), PUGNAc (*O*-(2-acetamido-2-deoxy-d-glucopyranosyliden)amino-*N*-phenylcarbamate), iNOS (inducible nitric oxide synthase), IL-6 (interleukin-6), TNF-α (tumor necrosis factor alpha), NF-κB (nuclear factor kappa light chain enhancer of activated B cells), COX2 (cyclooxygenase-2).

There is evidence to support *O*-GlcNAc modification of target proteins in the vasculature as a novel anti-inflammatory and vasoprotective mechanism [47,48]. Xing et al. [47] acutely treated ovariectomized rats with GlcN and PUGNAc before balloon injury of the right carotid artery and found that GlcN and PUGNAc each upregulated protein *O*-GlcNAcylation and significantly ameliorated acute inflammation [47]. Also, increased levels of proinflammatory HIS48^+^ granulocytes and ED1^+^ monocytes in response to injury were significantly reduced by GlcN and PUGNAc treatment through reduced infiltration into injured arteries [47]. The authors were also able to demonstrate that chronic (14 days) treatment with GlcN significantly reduced neointima formation in injured arteries compared with vehicle controls [47]. A protective anti-inflammatory role for *O*-GlcNAcylation in the vasculature is also supported by Hilgers et al. [48], who demonstrated that acute increases in protein *O*-GlcNAcylation were associated with reduced TNF-α-induced hypocontractility of rat aortic rings. In this study, iNOS protein expression was increased in TNF-α-treated rings, and this was attenuated by pretreatment with either GlcN or Thiamet-G, suggesting that acute increases in protein *O*-GlcNAcylation ameliorated TNF-α induced vascular dysfunction at least in part by limiting iNOS expression (Figure 4) [48].

Furthermore, Hwang et al. [68] proposed that GlcN could be a novel neuroprotective or anti-inflammatory agent. In this study [68], administration of GlcN was found to reduce infarct volume and ameliorates motor impairment and neurological deficits in a rat middle cerebral artery occlusion model of ischaemic stroke. Also, GlcN suppressed LPS-induced upregulation of pro-inflammatory mediators in BV2 microglial cells and RAW264.7 macrophages [68]. GlcN is a substrate for GFAT, the rate-limiting enzyme in the HBP (Figure 1). Increase in the influx of glucose and glutamine would result in increased UDP-GlcNAc levels and upregulation of *O*-GlcNAcylation. However, in this case, Hwang et al. [68] suggest that GlcN inhibits the *O*-GlcNAcylation of NF-κB, probably by disturbing the association between OGT and NF-κB.

In addition, Zou et al. [69] showed that increased *O*-GlcNAcylation by PUGNAc administration following induction of trauma-haemorrhage (TH) in fasted rats improves recovery of organ perfusion and function. In this study [69], they demonstrated that PUGNAc attenuated the TH-induced increase in plasma IL-6 levels, and suggested that PUGNAc, like GlcN, improves cardiac function and organ perfusion by reducing the level of circulating IL-6 and TNF-α after TH. The same researchers have also shown that treatment of fasted rats with GlcN attenuated TH-induced increases in NF-κB activation and reduced cardiac levels of TNF-α and IL-6 mRNAs, ICAM-1 protein and myeloperoxidase activity (Figure 4) [70]. These results demonstrate that the modulation of *O*-GlcNAc levels regulates activation of the NF-κB pathway in the heart, which may contribute to the observed GlcN-mediated improvement in cardiac function following haemorrhagic shock.

Also, sepsis leading to multiple organ damage is mainly caused by an uncontrolled systemic inflammatory response. However, the mechanisms responsible for this are unclear and our understanding of its pathogenesis remains incompletely understood [71,72]. As it shares common major blood lineages [73] and strikingly similar immune system architecture with humans, the zebrafish has been proposed as a tractable model to investigate the underlying pathophysiology of infectious diseases as well as immune and inflammatory disorders [73,74,75]. In addition, Hwang et al. [76] have demonstrated that pre-treatment of mice with GlcN upregulated protein *O*-GlcNAcylation and improved survival in the caecal ligation and puncture (CLP)-induced sepsis, and attenuated LPS-induced septic lung injury and systemic inflammation. In this study [76], they demonstrated that LPS reduced *O*-GlcNAcylation of nucleocytoplasmic proteins in liver, lung, and spleen. Also, LPS-induced downregulation *O*-GlcNAcylation in mouse lung was inhibited by GlcN, and the OGA level increased by LPS was suppressed by GlcN [76].

Another study has provided evidence that GlcN stimulates the *O*-GlcNAcylation of both nuclear and cytosolic forms of c-Rel [77]. Furthermore, this study demonstrated that upon stimulation with LPS, GlcN inhibits the binding of c-Rel to the NF-κB site in the iNOS promoter [77]. Although the mechanism through which GlcN exacts these effects is not fully understood, however, it is suggested that GlcN is impairs the interaction between OGT and c-Rel [77]. It has been observed in N9 microglial cells treated with LPS, the enhanced expression of iNOS, NO and ROS is mediated via the downregulation of OGT and protein *O*-GlcNAcylation, or via the upregulation of MAPKs phosphorylation and NF-κB translocation [78]. Also, overexpression of OGT inhibits LPS-induced activation of NF-κB and iNOS through modulation of histone acetylation either directly or indirectly [79]. Furthermore, Thiamet G improved clinical outcomes and ameliorated the neurological deficits when used to treat mice either before or after middle cerebral artery occlusion (MCAO) [80]. Additionally, the number of Iba1^+^ cells in MCAO mice were reduced when treated with Thiamet G [80]. Also, expression of MI markers was decreased by Thiamet G, conversely, the expression of the M2 markers increased in vivo [80]. Also, Thiamet G decreased the expression of iNOS and COX2 mainly by suppressing NF-κB p65 signaling (Figure 4) [80]. These results suggest that Thiamet G exerts a neuroprotective effect which suggest its usefulness as a potential anti-inflammatory agent for ischemic stroke therapy.

Furthermore, the deubiquitinase A20 has been identified as suppressor of NF-κB pathway activation [81]. Our understanding of the downstream regulation of the activities of A20 is still unclear. However, it has been shown that GlcN and Thiamet G which significantly increased *O*-GlcNAc modification of A20 enhanced its binding to TAB1, a key regulator of A20 activity [81]. These studies [81,82] suggest that *O*-GlcNAcylation is a critical regulatory modulator of the activities of A20, which consequently downregulates NF-κB signaling, including in vascular smooth muscle cells. Furthermore, it has been shown that the activities of Glutathione peroxidase 1 (GPX1) is induced by hyperglycaemia [83]. GPX1 is an anti-oxidant enzyme that is critical for cell survival. Hyperglycaemia enhances the *O*-GlcNAc modification of GPX1 and subsequently increases the association between non-receptor tyrosine kinase c-Abl and Arg in rat vascular smooth muscle cells [83]. Also, 1,2-dideoxy-2′-propyl-alpha-d-glucopyranoso-[2,1-d]-Delta2′-thiazoline (an OGA inhibitor) was shown to cause the activation of GPX1 in the mouse liver [83]. Furthermore, Hwang et al. [84] after stimulating with LPS, discovered that GlcN relieves the basal transcription activity of RNA polymerase II (RNAPII), a multiprotein complex that catalyzes the transcription of all protein-coding genes and several non-coding RNAs. They suggest that this observation was due to increased *O*-GlcNAcylation of RNAPII and DNA binding upon treatment with GlcN, which are inhibited by LPS [84].

### 3.2. Intrinsic Role of OGT and OGA in Inflammatory Pathways

Findings showing the intrinsic involvement of the key modulating enzymes of *O*-GlcNAcylation in inflammation are beginning to emerge [46,85,86,87]. Mutual and balanced interaction between OGT and OGA is critical for normal *O*-GlcNAc homeostasis. However, sustained alteration in form of enhancement or inhibition of these key enzymes initiates the development of *O*-GlcNAcylation mediated pathologies with inflammatory components. Recent findings have demonstrated that OGT promotes inflammatory responses in macrophages [46,49,85,86,88]. NF-κB which is a critical regulator of pro-inflammatory responses in macrophages has been shown to be *O*-GlcNAc modified [49]. Allison et al. [49] demonstrated that OGT could co-localise to NF-κB–regulated promoters causing the modification of Thr305 residue of the NF-κB subunit RelA. This was shown to promote acetylation on K310 of RelA, and further enhanced NF-κB transcriptional activity after stimulation of tumour necrosis factor (TNF) [49]. Allison et al. [49] further showed that a knockdown of OGT abolishes p300-mediated acetylation of RelA on Lys310, a posttranslational mark required for full NF-κB transcription. Similarly, Ramakrishnan et al. [85] identified Ser350 as the site of modification and activation of NF-κB subunit c-Rel. They also showed that preventing the OGT mediated *O*-GlcNAc modification of this residue repealed c-Rel–mediated expression of the cytokine-encoding genes *IL2*, *IFNG*, and *CSF2* in response to TCR activation, whereas increasing the extent of *O*-GlcNAcylation of cellular proteins promoted the expression of these genes [85].

Microglial cells are the brain resident macrophage [89]. In BV2 microglia cells, c-Rel has been shown to interact with OGT upon lipopolysaccharide (LPS) treatment, which promotes c-Rel *O*-GlcNAcylation and formation of a c-Rel-p50/p105 heterodimeric complex [77]. More so, this study [88] demonstrated that the S-nitrosylation of OGT inhibits its catalytic activity in resting RAW 264.7 murine macrophage cells [88]. However, stimulation of OGT with LPS results in de-nitrosylation of OGT which then enhanced OGT-mediated *O*-GlcNAc modification of p65/RelA [88]. Furthermore, attenuation of *O*-GlcNAcylation negatively modulates p65 nuclear translocation, resulting in diminished production of nitric oxide (NO) and IL-1β [88]. Also, the role of STAT3, a critical transcription factor in promoting inflammation and tissue repair has been well enumerated [90,91]. More so, STAT3 can induce IL-10 production in macrophages to suppress inflammation [92]. It has been previously demonstrated that OGT modulated *O*-GlcNAc modification of STAT3 at transactivation domain Thr717 competitively downregulates STAT3 phosphorylation which results in reduced production of IL-10 [46]. Furthermore, in macrophages derived from the bone-marrow, OGT expression can be transcriptionally downregulated by myeloid-derived cullin 3 (CUL3), a process dependent on nuclear factor-2 (Nrf2) which binds to the *Ogt* promoter to increase gene transcription [46,93,94]. Therefore, CUL3 counteracts STAT3 *O*-GlcNAcylation to elevate STAT3 phosphorylation and inhibit inflammation [46].

In contrast, many studies have demonstrated that increased *O*-GlcNAcylation correlates with suppression of inflammation in sepsis and ischemia [76,95,96,97,98,99]. Upon GlcN treatment, which bypasses the GFAT rate-limiting step to induce hyper-*O*-GlcNAcylation [95], the transcriptional activity of c-Rel is inhibited, thereby reducing NF-κB-mediated expression of inducible nitric oxide synthase (iNOS) in LPS stimulated BV2 microglial cells [68,77]. Furthermore, it has recently been shown that receptor-interacting serine/threonine-protein kinase 3 (RIPK3) can be O-GlcNAc modified [96]. RIPK3 is a member of the necrosome complex, a complex consisting of RIP1, RIP3, and Fas-associated protein with death domain causing rapid plasma membrane rupture and induction of an inflammatory response. This is done through the release of damage-associated molecular patterns and cytokines [97,98]. Also, RIPK3 reduces inflammation by diminishing the production of pro-inflammatory cytokines resulting from necroptosis of macrophages [100]. Interestingly, *O*-GlcNAc modification of RIPK3 at Thr467 is believed to suppress RIPK3-RIPK1 and RIPK3-RIPK3 complex formation, therefore preventing necroptosis of macrophages [96]. *Ogt^f/f^ Lyz2*-cre conditional knockout mice, in which *Ogt* is specifically deleted in macrophages, revealed significantly increased activation of RIPK3, elevated production of inflammatory cytokine, and more severe mortality in experimental sepsis when compared with control mice [96]. Furthermore, Yang et al. [87] have shown that OGT prevents macrophage mediated inflammation and metabolic disturbance by suppressing the phosphorylation of S6 kinase beta-1 (S6K1). Phosphorylation of S6K1, a ribosomal protein is critical for macrophage proinflammatory activation [87]. More so, OGT mediated O-GlcNAcylation is downregulated during macrophage proinflammatory activation, and knockout of OGT in high fat-diet induced obese mice enhances macrophage proinflammatory polarization and promotes adipose tissue inflammation [87].

On the other hand, acute upregulation of *O*-GlcNAcylation by inhibiting OGA have been shown to prevent inflammation-induced vascular dysfunction, hence, confer a protection on the heart and vasculature [48]. Hilgers et al. [48] demonstrated that acute increase in *O*-GlcNAcylation by inhibiting OGA prevented TNF-α-induced vascular dysfunction. Furthermore, *O*-GlcNAcylation can possibly modulate the polarisation of M2 macrophages in a pattern that contribute to resolution of inflammation and tissue repair [76,80,99]. This study [76] demonstrated that treatment with GlcN reduced M1 macrophage-specific gene expression profiles in macrophages in an LPS-induced septic lung injury animal model. More so, when Thiamet-G, a selective OGA inhibitor [80] was administered to a mouse model of middle cerebral artery occlusion, there was an increase in the expression M2-specific markers in microglia [80]. Also, there was suppression of NF-κB p65 signaling which resulted in diminished expression of iNOS and cyclooxygenase-2 (COX-2) expression [80]. This strongly suggests that alteration *O*-GlcNAc homeostasis can vary the differentiation of M2 versus M1 macrophages in tissues.

## 4. Targeting *O*-GlcNAcylation in Inflammatory Derangements: Perspectives

*O*-GlcNAcylation is currently an unexplored therapeutic target in the management of pathologies with inflammatory derangements; this may be due to the fact that the involvement of this dynamic PTM is just beginning to evolve. Also, our current understanding of the involvement of *O*-GlcNAcylation in inflammatory pathways is unclear. More so, that *O*-GlcNAcylation seem to be a dual-directional regulator of inflammatory system. Therefore, further characterisation would help improve our current knowledge which could pave way for the development of new therapeutics that can find their way into mainstay clinical applications. Over 5000 proteins have now been identified as targets for *O*-GlcNAcylation and this will continue to increase due to technological advances in the mapping of protein *O*-GlcNAcylation sites [22,100,101]. As we have described in detail, the role of *O*-GlcNAcylation in the activation of pro-inflammatory and immunomodulating pathways has gained more attention. For example, findings have shown that *O*-GlcNAcylation activates NF-κB and NFAT pathway [39,50,51,52,102]. Activation of NF-κB initiates the transcription of relevant downstream target genes with pro-inflammatory tendencies and on the other hand, activation of the NFAT pathway has been linked with modification of proteins in human bronchial epithelial cells by fibroblast growth factor 23 resulting in upregulation of IL-6 and regulation of airway inflammation [103].

Furthermore, recurring and/ prolonged inflammation, a common and characteristic feature of a variety of disorders such as autoimmune disease, diabetes, and cancer have been attributed to aberrant regulation of NF-κB activity [104,105,106]. Therefore, a clearer understanding of the mechanisms that underpin the involvement of NF-κB in the inflammatory process may have great biological and clinical significance. Increased cellular *O*-GlcNAc level which is typical in diabetes and insulin resistance is usually accompanied by NF-κB activation, and treatments that upregulate *O*-GlcNAcylation appear to have anti-inflammatory and pro-survival effects during acute injuries like myocardial infarction, burns, trauma and sepsis [69,70,107,108]. For instance, GlcN and PUGNAc improved organ perfusion and function after trauma-hemorrhage in fasted male rats [69]. Also, Xing et al. [109] showed that treatment of rat aortic smooth muscle cells with GlcN and PUGNAc prevented TNF-α induced inflammatory stress by upregulation of *O*-GlcNAc modification of p65 on Ser536. Xing et al. [109] further suggested that GlcN and PUGNAc inhibited TNF-α induced phosphorylation of NF-κB p65, thus inhibiting NF-κB signaling in rat aortic smooth muscle cells. In vivo, dextran sodium sulfate-induced phosphorylation of NF-κB p65 and IL-1β mRNA expression are significantly lower in *Ogt*-transgenic when compared wild type mice. This suggests that acute colitis could be prevented by upregulating *O*-GlcNAcylation which result in diminished acute inflammation [110].

Also, alteration in the metabolic pathways of effector T cells is core to the progress of insulin resistance and atherosclerosis and this may lead to enhanced supply of metabolites to the HBP, thereby promoting *O*-GlcNAc modification. More so, effector T cells such as Th1 and Th17 cells are crucial for several autoimmune diseases, including inflammatory bowel disease, rheumatoid arthritis, multiple sclerosis (MS), and systemic lupus erythematosus (SLE) [111]. It has been suggested that the progression of SLE is enhanced by the reactivation of the silenced X-chromosome due to CD4^+^ T cell DNA demethylation and diet [112]. Hence, it is proposed that the magnitude of overexpression of OGT in CD4^+^ T cells could be a critical factor that contributes to the progression of SLE in women [112]. Additionally, it has been demonstrated that miR-15b suppressed the differentiation of Th17 cells which are likely the most critical pathogenic factor of human MS [113]. It is suggested that this results in suppression of the pathogenesis of MS by decreasing the expression of OGT in an NF-κB p65- and c-Rel-dependent manner [113]. Taken together, these observations should lead to the investigation of the links between protein *O*-GlcNAcylation and the activation of T cells in metabolic and autoimmune diseases. We believe that further characterisation of the role of OGT and *O*-GlcNAc modification in autoimmunity may yield new therapeutic targets for autoimmune diseases with inflammatory components.

Furthermore, the involvement of *O*-GlcNAcylation in pro- versus anti-inflammatory processes is dependent on the distinct GlcN stimulus which regulates inflammation by sensing both a healthy nutritional status and overnutrition. At normoglycaemic concentrations, GlcN enhances LPS-triggered inflammation in macrophages in a dose-dependent manner, conversely, in high glucose cell culture the inflammation of macrophages was suppressed [114]. Also, it was observed that LPS-stimulated induction of iNOS as well as an increase in DNA binding of c-Rel to the iNOS promoter in macrophage cells is increased by GlcN in normoglycaemic conditions and decreased in high glucose conditions [114]. In addition, there is a high risk in pregnancy due to hyperglycaemia which could have negative effects on the fetus. Considering that *O*-GlcNAcylation is a nutritionally responsive modification; hence, excess flux of glucose may alter the *O*-GlcNAc homeostasis in the intrauterine environment resulting in metabolic deregulation of the offspring [115]. Furthermore, inhibition of TNF-α and IL-8 gene expression in osteoarthritis is mediated in part by GlcN-induced *O*-GlcNAcylation [116]. Therefore, these findings suggest that depending on the cell state, the nature of insult, and the cellular nutrition state, HBP may swiftly adapt the management pattern to regulate inflammation, resulting in either pro- or anti-inflammatory outcomes [114]. Thus, proper modulation of *O*-GlcNAc homeostasis presents a viable therapeutic strategy for combating inflammatory diseases and metabolic dysregulation such as sepsis, diabetes, and osteoarthritis.

Pharmacological modulation of the critical enzymes (OGT and OGA) that regulate this dynamic PTM might be a critical lead way in treating inflammatory derangements. Drugs like alloxan [117,118], benzoxazolinones [119], BADGP (Benzyl-2-acetymido-2-deoxy-α-d-galactopyranoside) [120,121,122] are used as OGT inhibitors but due to off-target effects and toxicities, their role over the years has been limited to experimental and in vitro studies [122,123]. However, more recently developed OGT inhibitors such as Ac-5SGlcNAc [124,125], OSMI 1 [126], OSMI 2-4 [127], and L01 [128] have not only helped improve the understanding and characterisation of this dynamic PTM but are more promising as potential pharmacological agents as they have shown improved specificity and encouraging pharmacodynamic profiles [122]. On the other hand, development of therapeutic agents used clinically to increase *O*-GlcNAcylation level has been more successful [122]. These agents include OGA inhibitors such as Thiamet-G (5H-Pyrano[3,2-d]thiazole-6,7-diol2-(ethylamino)-3a,6,7,7a-tetrahydro-5-(hydroxymethyl)-(3aR,5R,6S,7R,7aR)), PUGNAc (*O*-(2-acetamido-2-deoxy-d-glucopyranosyliden)amino-*N*-phenylcarbamate), NButGT (1,2-dideoxy-2′-propyl-α-d-glucopyranoso-[2,1-d]-Δ2′-thiazoline), and more recently developed GlcNAcstatin [122,129,130]. Also, glucosamine, which increases the UDP-GlcNAc concentrations by bypassing GFAT [95,122].

However, considering that OGA and OGT are ubiquitously expressed, modulation of their expression with drugs could come with numerous adverse drug reactions. Also, considering that the use of OGA and OGT global knockout mice is not viable as this usually result in high mortality [131,132,133,134]. Therefore, cell-specific knock out of these key enzymes to fully validate the role of O-GlcNAc modification in inflammatory responses in key cell types in animal models of disease would inform studies in human. Also, assessment of the expression of OGT/OGA change in target cell types in disease and determining if targeting OGA/OGT is limited to catalytic inhibitors targeted at their active sites could be characteristic. In view of this, we believe it is viable to target OGT/OGA-target protein interaction with protein-protein interaction inhibitors [135,136]. Having identified the sites of interaction, it is possible to design and optimize a blocking peptide and use this as a basis to screen for drug-like compounds that function as peptidomimetics [135,136].

## 5. Conclusions

Findings highlighted in this review have shown that the involvement of *O*-GlcNAcylation in the modulation of inflammatory pathways is complex, wide-ranging and substantial, yet it is not a populartarget for drug development in the management of inflammatory pathologies. Therefore, we propose that targeting *O*-GlcNAcylation is a viable therapeutic target for future management of pathologies characterised by dysregulation of inflammatory pathways.
